# Socioeconomic Indicators Are Independently Associated with Nutrient Intake in French Adults: A DEDIPAC Study

**DOI:** 10.3390/nu8030158

**Published:** 2016-03-10

**Authors:** Wendy Si Hassen, Katia Castetbon, Philippe Cardon, Christophe Enaux, Mary Nicolaou, Nanna Lien, Laura Terragni, Michelle Holdsworth, Karien Stronks, Serge Hercberg, Caroline Méjean

**Affiliations:** 1 U1153 National Institute of Health and Medical Research, U1125 National Institute for Agricultural Research, National Conservatory of Arts and Crafts, Nutritional Epidemiology Research Team (EREN), Sorbonne Paris Cité, Paris 13 University, Paris 7 and 5, Bobigny 93017 Cedex, France; s.hercberg@eren.smbh.univ-paris13.fr (S.H.); c.mejean@eren.smbh.univ-paris13.fr (C.M.); 2 Department of Chronic Diseases and Injuries, French Institute for Health Surveillance, Nutritional Epidemiology and Surveillance Unit, Sorbonne Paris Cité, Paris 13 University, Bobigny 93017 Cedex, France; Katia.Castetbon@ulb.ac.be; 3 School of Public Health, Université Libre de Bruxelles (ULB), Bruxelles B-1070, Belgium; 4 National Institute for Agricultural Research, UR 1303 ALISS, Ivry sur Seine 94200, France; Philippe.Cardon@ivry.inra.fr; 5 Lille 3 University—EA CeRies, Villeneuve-d’Ascq 59650, France; 6 University of Strasburg, UMR LIVE 7362, Strasbourg 67000, France; christophe.enaux@live-cnrs.unistra.fr; 7 Department of Public Health, Academic Medical Centre, University of Amsterdam, Amsterdam 1100DD, The Netherlands; m.nicolaou@amc.uva.nl; 8 Department of Nutrition, Faculty of Medicine, University of Oslo, Oslo 0316, Norway; nanna.lien@medisin.uio.no; 9 Department of Nursing and Health Promotion, Faculty of Health Sciences, Oslo and Akershus University of Applied Sciences, Oslo 0130, Norway; laura.terragni@hioa.no; 10 Public Health section, School of Health and Related Research (ScHARR), The University of Sheffield, Sheffield, Sheffield S1 4DA, UK; michelle.holdsworth@sheffield.ac.uk; 11 Department of Public Health, Academic Medical Center, University of Amsterdam, Amsterdam 1100 DD, The Netherlands; k.stronks@amc.uva.nl; 12 Department of Public Health, Avicenne Hospital, Bobigny 93300, France

**Keywords:** socioeconomic position, nutrient intake, education, income, occupation, inequalities

## Abstract

Studies have suggested differential associations of specific indicators of socioeconomic position (SEP) with nutrient intake and a cumulative effect of these indicators on diet. We investigated the independent association of SEP indicators (education, income, occupation) with nutrient intake and their effect modification. This cross-sectional analysis included 91,900 French adults from the NutriNet-Santé cohort. Nutrient intake was estimated using three 24-h records. We investigated associations between the three SEP factors and nutrient intake using sex-stratified analysis of covariance, adjusted for age and energy intake, and associations between income and nutrient intake stratified by education and occupation. Low educated participants had higher protein and cholesterol intakes and lower fibre, vitamin C and beta-carotene intakes. Low income individuals had higher complex carbohydrate intakes, and lower magnesium, potassium, folate and vitamin C intakes. Intakes of vitamin D and alcohol were lower in low occupation individuals. Higher income was associated with higher intakes of fibre, protein, magnesium, potassium, beta-carotene, and folate among low educated persons only, highlighting effect modification. Lower SEP, particularly low education, was associated with lower intakes of nutrients required for a healthy diet. Each SEP indicator was associated with specific differences in nutrient intake suggesting that they underpin different social processes.

## 1. Introduction

Socioeconomic health inequalities have been widely described in the literature, in terms of morbidity and mortality [1,2,3,4]. Diet appears to substantially contribute to these socioeconomic differences in mortality (up to 66%) [5,6,7]. An important body of literature has concluded that a high socioeconomic position (SEP) is consistently associated with healthy dietary patterns including a greater consumption of fruits, vegetables, and whole-grain foods, whereas individuals of low SEP are more likely to consume more refined cereals, fatty meat and added fats [8,9,10]. Compared with studies on food groups, literature on the relationship between SEP and nutrient intake is scarcer and, to our knowledge, very few studies have used recent data on this particular topic. Previous studies have highlighted that low SEP groups have lower intakes of fibre, minerals such as calcium and vitamins, e.g., vitamin C and folate [8,11]. According to a review published in 2008, results about the association of energy and macronutrient intake with SEP are equivocal. In particular, a socioeconomic gradient in total fat intake has not been consistently observed [8].

Although some studies regarding socioeconomic disparities in nutrient intake have been conducted, very few have used all three major SEP indicators (education, occupation and income) to explore the relationship between SEP and nutrient intake or have investigated the interaction between all of these SEP indicators [12,13,14,15]. Although education, income and occupation are correlated, they are not interchangeable and can even have additive or synergistic effects on food intake [13,14,16]. The fact that the correlations between the SEP indicators are generally modest suggests some shared association but also their unique role [16]. In fact, education is linked to diet through knowledge and attitudes while income reflects financial means and occupation can represent one’s social network [17]. Hence using only one indicator of SEP can lead to misinterpretations of the differences in intake between socioeconomic groups [13,15]. As they might affect diet differently, to study the independent role of each SEP indicator and their interaction is useful for a better understanding of the socioeconomic inequalities in the nutrient quality of the diet.

Within the Determinants of Diet and Physical Activity (DEDIPAC) joint action of the European Joint Programming Initiative “A Healthy Diet for a Healthy Life”, providing frameworks of determinants of dietary behaviors and social inequalities is a key objective [18]. The aim of this study was to investigate the independent association of three SEP indicators (education, occupation and income) with nutrient intake in French adults participating in the NutriNet-Santé study. In addition, as income is determined by education and occupation, the effect modification of these SEP indicators on the relationship between income and nutrient intake was assessed.

## 2. Materials and Methods

Subjects were participants in the NutriNet-Santé study, a large web-based prospective observational cohort launched in France in May 2009. It was implemented in the French general population targeting internet-using adult volunteers aged ≥18 years. The study was designed to investigate the relationships between nutrition and health, as well as the determinants of dietary behaviours and nutritional status. The design, methods and rationale have been described previously [19]. Briefly, participants were included in the cohort once they completed a baseline set of questionnaires assessing dietary intake, physical activity, and socioeconomic and health status. At follow-up, participants completed the same set of questionnaires every year. Additionally, each month they were invited to fill out complementary questionnaires related to determinants of food behaviours, nutritional and health status.

This study was conducted according to guidelines laid down in the Declaration of Helsinki and all procedures were approved by the Institutional Review Board of the French Institute for Health and Medical Research (IRB Inserm No. 0000388FWA00005831) and the French Data Protection Authority (Commission Nationale Informatique et Libertés No. 908450 and No. 909216). Electronic informed consents were obtained from all participants.

### Data Collection

#### Dietary Intake

At baseline, participants were invited to complete 3 non-consecutive validated web-based 24-h dietary records, randomly assigned over a 2-week period (2 weekdays and 1 weekend day) [19,20,21]. The dietary record was completed via an interactive interface and was designed for self-administration on the Internet [22]. The web-based dietary assessment method relies on a meal-based approach, recording all foods and beverages (type and quantity) consumed at breakfast, lunch, dinner and all other eating occasions. Participants estimated portion sizes for each reported food and beverage according to standard measurements (e.g., home containers, grams indicated on the package) or using validated photographs [23]. The values for energy, macronutrients and micronutrients were estimated using published nutrient databases [24] and completed for recent market foods and recipes. For each participant, daily mean quantities of nutrients were calculated from 24-h records, weighted according to the day (weekdays or weekend). As exclusion criterion in our sample, energy-under-reporting participants were identified using the method proposed by Black, which allows comparing energy intake to the basal metabolic rate (BMR) taking into account the physical activity level (a physical activity level of 1.55 was used to identify underreporting participants, while a value of 0.88 was used for extreme underreporting participants) [25]. BMR was estimated using Schofield equations according to sex, age, weight and height collected at enrolment in the study [26]. In addition, participants had the option to indicate whether the reported consumption was representative of their usual diet or considerably differed (due to illness, dieting, a social event, *etc.*) and that information was taken into account to identify specific conditions that could objectively explain low energy intake. In this case, subject was not excluded from the study population.

#### Socioeconomic Position (SEP)

Socioeconomic and demographic data were collected at baseline using a web-based self-completed questionnaire previously validated [27]. The highest attained diploma was collected to assess educational level using referential of INSEE (French National Institute of Statistics) [28]. The four following categories were used: primary education, secondary education, undergraduate (corresponding to up to 3 years after high school diploma), and postgraduate (>3 years after high school diploma). Occupation was assessed using the definition of INSEE [29] and was recoded into 6 classes: never employed (homemakers, students, and disabled), manual workers, employees, intermediate professions (e.g., technicians, skilled employees, teachers, nurses), self-employed (craftsman, shopkeeper, company manager and farmer) and managerial staff. Unemployed and retired individuals were asked to indicate their last occupation status. In this particular case, the previous status was used to assess the occupation in the analyses. Participants were asked about monthly household income including total salary, social benefits, family allowance and rental income. Subjects could also choose not to indicate their household income. The monthly household income was calculated according to household composition, reported by the participant. Thus, reported monthly household income was divided by the number of household units (HU), *i.e.*, 1 HU for the first adult in the household, 0.5 HU for other persons aged ≥14 years and 0.3 HU for children <14 years [30]. The following five categories of monthly income were used: <1200 €, 1200–1800 €, 1800–2700 € and >2700 € per HU and a category grouping individuals who refused to answer.

#### Statistical Analysis

The present analysis focused on participants included in the NutriNet-Santé cohort study between May 2009 and October 2013, living in the mainland France, who completed at least three 24-h dietary records at baseline, who did not under-report energy, and who had provided data on education level, occupation and income.

All analyses were performed separately for men and women since sex interactions were significant. Associations between nutrient intake and the three SEP indicators (education, income and occupation) were assessed by using analysis of covariance. The three indicators were included simultaneously in the models in order to assess their independent effect. All models assessing associations between nutrient intake and SEP indicators were adjusted for age and total energy intake. Collinearity between the three SEP indicators was investigated by examining the variance inflation factors. Relative difference in intake between individuals belonging to the highest SEP category and those of the lowest SEP category was computed as: ((mean intake of the highest SEP category − mean intake of the lowest SEP category)/(mean intake of the highest SEP category)) × 100. A positive relative difference shows higher intake of nutrient in the highest SEP category and a negative relative difference shows lower intakes in the highest SEP category. Interactions between income and education or occupation and between education and occupation were also tested. When interactions were significant (*p*-value < 0.05), we performed analyses of associations between nutrient intake and income, stratified by education or occupation strata and adjusted for age and total energy intake. Because the large sample size increases the likelihood of significant statistical tests, results were interpreted as significant only with a specific P-value and when relative difference was >5%. We considered that this difference represents a difference in daily intake that could have long-term consequences on health. A *p*-value < 0.05 was first considered statistically significant. Then, to take into account multiple comparisons, we calculated the Bonferroni correction for each model, leading to a *p*-value < 0.002 in non-stratified models, a *p*-value < 0.01 in stratified models by education and a *p*-value < 0.0083 in stratified models by occupation. Regarding to occupational categories, comparisons between intakes of self-employed and never-employed subjects and those of the other occupational categories were not interpreted, since these two groups are very heterogeneous in terms of social status and networks, although they were included in the multivariate analysis. In addition, individuals who refused to declare their income had very diversified sociodemographic profiles, so we did not interpret comparisons between their intake and those of the other income classes. Data pre-treatment and statistical analyses were performed using SAS (version 9.3; SAS Institute, Inc., Cary, NC, USA).

## 3. Results

### 3.1. Description of the Study Population

Among 123,269 participants included between May 2009 and October 2013, we excluded 17,344 individuals (14.1%) who had not provided three 24-h records, 13,306 underreporting subjects (10.8%) and 719 participants with missing data for at least one of the three socioeconomic factors (0.6%), thus leaving to 91,900 individuals for analysis (72,154 women and 19,746 men). Percentages of young subjects (18–30 years), individuals with undergraduate educational level, employees and never employed persons, those belonging to the lowest income class were higher in women than in men while percentages of elderly (>65 years), subjects with post-graduate education, managerial staff, manual workers and self-employed, and individuals belonging to the highest income class were lower (Table 1).

### 3.2. Analysis of the Collinearity of the SEP Indicators

Overall, variance inflation factor of each SEP indicator was between 1.04 and 1.63, showing that SEP indicators were not collinear. Only those results for which the difference in mean intake between participants belonging to the highest SEP category and those of the lowest category was >5% are described.

### 3.3. Associations between Nutrient Intakes and SEP Indicators

In both sexes, no significant association was found between SEP and intake of iron, sodium, calcium, vitamin A, vitamin E, total carbohydrates, lipids, polyunsaturated fatty acids, monounsaturated fatty acids and saturated fatty acids, regardless of the SEP indicator (Table 2 and Table 3).

#### 3.3.1. Education

In both sexes, individuals with postgraduate education reported lower protein intake (−5%) and lower cholesterol intake (difference: −9% in men and −7% in women) and higher intakes of fibre (around +7%), beta-carotene (+6% to +9%) and vitamin C (+7% to +9%) than those in the lowest education category (Table 2 and Table 3). Women with postgraduate education reported higher total energy intake than those with primary education (difference: +5.8%). Men with postgraduate education consumed more sugars (+6%) than men with primary education (Table 3).

#### 3.3.2. Income

In men and women, individuals with the highest income reported lower intake of complex carbohydrates (−6%) and higher intake of folate (+5%), vitamin C (+12% to +14%) and magnesium (+6%) than those in the lowest category of income (Table 2 and Table 3). Regarding potassium, men with the highest income had higher intake than individuals with the lowest income (+5%) (Table 3).

#### 3.3.3. Occupation

Managerial staff participants had higher intake of alcohol (+ 15.6% in women and +8.6% in men) (Table 2 and Table 3). Female managerial staff had higher magnesium (+6.5%), vitamin D (+11.5%) and beta-carotene (+7%) intakes than female manual workers (Table 2).

### 3.4. Associations between Income and Nutrient Intake Stratified by Education or Occupation Levels

#### 3.4.1. Stratified Analyses by Education

In both sexes, interaction between income and education was significant for beta-carotene (*p* = 0.0001 in women and *p* = 0.03 in men), folate (*p* < 0.0001 in women and *p* = 0.008 in men) and magnesium (<0.0001 in both sexes). In both sexes, in primary and/or secondary education levels, individuals with highest income had higher intakes in beta carotene, folate and magnesium than those with the lowest income (differences: +6% to +13%) (Table 4 and Table 5) while no difference according to income was found in the highest category of education. In men, significant interactions between education and income were found regarding proteins *(p* = 0.01) and fibre (*p* = 0.0003) and potassium (*p* = 0.002). In men, difference > 5% was found only in individuals with primary education. In this group, individuals belonging to the highest income category had higher protein intake than those in the other income classes (Table 5). Only in secondary education level, men with the highest income had higher fibre intake than those with the lowest income (difference: +6.3%) (Table 5). Only in individuals with primary and secondary education, men with the highest income had higher potassium intake than subjects belonging to the two lowest income classes (differences: +5% to +6.5%) (Table 5).

#### 3.4.2. Stratified Analyses by Occupation

In both sexes, interaction between income and occupation was significant for magnesium (*p* = 0.04 in women and *p* = 0.0499 in men). Employees with highest income consumed more magnesium than those of other income categories (differences: +5% to +8%) (Table 6). In addition, only male managerial staff belonging to the highest income class had higher magnesium intake than men with lower income (+7.2%) (Table 6). In women, significant interaction between income and occupation for intake of complex carbohydrates was found (*p* = 0.0004). Stratified analyses showed that, only in the intermediate professions, individuals belonging to the highest income class consumed less complex carbohydrates than subjects in the lowest income class (difference: −6%) (Table 6). In men, interaction between income and occupation was significant for potassium (*p* = 0.03) and alcohol (*p* = 0.01). In manual workers and employees, men with highest income had higher potassium intake than individuals of the other income categories (differences: +9% to +11% in manual workers and +5 to +6% in employees) (Table 6). In managerial staff, men belonging to the highest income class consumed more alcohol than individuals in the two lowest income classes (differences: +6% to +10%) (Table 6).

## 4. Discussion

The present study addressed differences in nutrient intake between low and high socioeconomic groups in French adults using education, income and occupation as indicators of the SEP. Each SEP indicator was associated with specific differences in nutrient intakes suggesting that they underpin different social processes. Particularly, our findings showed that low educated individuals tend to have lower intakes of nutrients required for a healthy diet. Indeed, low educated participants had higher intakes of protein and cholesterol and lower intakes of fibre, vitamin C and beta-carotene. Low income individuals had higher intakes of complex carbohydrates and lower intakes of magnesium, potassium, folate and vitamin C while intakes of vitamin D and alcohol were lower in low occupation individuals. No difference in the intake of lipids and fatty acids was found. In addition, education modulated the relationships between income and intakes of fibre, protein, magnesium, potassium, beta-carotene and folate. In low education levels only, higher income was associated with higher intake of these nutrients.

Our finding showing that lipids and fatty acids were not influenced by SEP was concordant with a previous literature review [8]. This lack of difference may be explained by very high intakes, above recommendations, whatever the SEP groups, suggesting that fat intake is influenced by other factors, such as high exposure to fatty foods in the food supply, that equally reach all SEP categories. Consistent with previous studies, our findings showed no relationship between overall carbohydrate intake and the three SEP indicators [8,13]. Unlike previous studies, our finding did not show any association between intake of calcium and iron and SEP [11,13,31,32]. A study conducted in French adults aged 45–60 years also showed no difference in intake according to SEP [33]. This result could be due to high intakes of calcium, whatever the SEP groups. A previous work has shown that calcium intake was satisfactory in a representative sample of French adults [34]. Consumption in all SEP groups of high amount of dairy products, the main contributor of calcium intake in France, could explain this high calcium intake. Among dairy products, milk was more consumed by manual workers than managerial staff and cheese consumption increased with education [8,33]. Further analyses on the socioeconomic differences in the consumption of dairy products in our sample could help us to understand why no statistical difference was found in calcium intake.

### 4.1. Education in Relation to Nutrient Intake

Regarding protein and cholesterol intakes, our finding showing an inverse educational association with higher intakes in low educated persons was concordant with previous studies [35,36,37,38]. In France, meat, poultry and processed meats represent 34% of total protein intake, followed by dairy products (17%), ready-to-eat foods (8%) and fish (6%) [39]. The contribution of animal proteins to total intake of highly educated individuals may be lower than in low educated groups because they are more likely to perceive the negative implications of animal products for health, therefore reducing their total intake of animal-based products. The fact that high educated participants consumed less cholesterol reinforces this hypothesis as cholesterol is mostly present in animal based products. The symbolic role of meat, such as its contribution to physical strength and energy, and social norms in low educated persons could also influence the decision to eat meat and maintain its important status in meals in low educated persons [40]. In other French studies [41,42], a low education was associated with a “meat products and alcohol” dietary pattern. In concordance with previous works, positive educational gradients in fibre, vitamin C and beta-carotene intakes were observed in our study [8,11,31,36,43]. Compared to low educated participants, high educated individuals may have an increased knowledge of diet-related health problems and may perceive the long term consequences of diet on health. High levels of education can be associated with better nutrition knowledge [44,45,46,47] and it has been shown that nutrition knowledge is associated with healthier food purchasing or healthy eating habits leading to consumption of food groups rich in nutrients such as vitamins and fibre [45,47]. It can also modify the association between SEP and diet [44,48]. Attitudes towards health benefits of diet and awareness of key messages may lead high educated persons to comply better with dietary recommendations [49] by consuming less animal products and more wholegrain foods and fruit and vegetables, the main sources of fibre in France and contributors to vitamin C and beta carotene intakes [8,10,44,49,50,51,52].

### 4.2. Income and Occupation in Relation to Nutrient Intake

Low income participants had lower intakes of magnesium, potassium, folate and vitamin C Income has an effect on available material resources and reflects financial accessibility to healthy foods [15,17]. Then, these lower intakes, particularly those of vitamins, in individuals with low income may be explained by a limited financial access to fruits and vegetables among this category, as they are the main source of folate and vitamin C in the French population [53,54,55]. An inverse relationship between the intake of complex carbohydrates and income was found. This could be due to the fact that low SEP groups consumed more bread and starchy foods, the main contributors of complex carbohydrates, than high SEP groups, probably because they are affordable [8,56,57]. Mean vitamin D intake was below recommendations in all SEP subgroups and mean intake of low occupation participants was weaker. Some of the strongest differences between occupation and income categories were observed regarding vitamin D or alcohol. The main source of vitamin D intake in the French population is fish (38% of the intake) [58]. This positive association between vitamin D intake and occupation could be explained by a higher proportion of fish consumers among high occupational subjects, which has been observed previously [32]. However, vitamin D status is determined weakly by dietary intake [59,60]. Our finding that alcohol intake was positively associated with occupation and income was concordant with previous studies [36,61]. Occupation is related to social standing and environmental conditions [17]. Social networks and professional relationships in particular influence the consumption of alcoholic beverages. Indeed, in social representations, consumption of alcoholic beverages might lead to more conviviality in the professional environment.

### 4.3. Effect Modification of Education and Occupation

Associations of intakes of fibre, folate, beta-carotene, magnesium, and potassium with income were significant only in low educational or occupational categories, suggesting that the barrier of the cost of foods containing high level of these nutrients, such as whole grain foods, fruits and vegetables, can be overcome by education or occupation. In individuals with primary or secondary education, income appears to be a key element of dietary intakes as increased income seems to improve the capacity to buy and then to consume more nutrient-dense foods, known as costly [57]. In subjects with high education or high occupation, no difference in nutrient intake was observed between income classes, suggesting that knowledge and beliefs of individuals with high education and occupation levels may lead them to act positively on their diet, thus consuming more healthy recommended foods regardless of income. Education may lead individuals to develop strategies helping them to cope with the financial barrier of nutrient-dense foods such as fresh fruits or vegetables [13].

### 4.4. Strengths and Limitations

Several limitations of our study should be taken into account. Since the NutriNet-Santé Study is a voluntary cohort, the sampling is not random and more subjects were women, belonged to high education and occupation groups and had a healthier lifestyle than the general population, with higher intake of fibre and calcium [34,62]. In particular, the overrepresentation of women in our sample could be explained by the fact that women are more likely to participate in voluntary-based health and epidemiological studies, whatever the field concerned [63]. They may also have increased interest in nutrition, compared to men. Although men represented only 21.5% of our sample, the distribution of men in the different SEP categories seems sufficient to interpret differences in intake between these categories. In addition, the web-based design might not increase, but possibly even mitigate recruitment biases [64]. Indeed, a previous work regarding participants in our cohort showed that the exclusive use of the Internet for data collection and follow-up may help to increase the proportion of population groups which are often underrepresented in volunteer cohorts such as men and older subjects [65]. In addition, previous work showed a great geographic and socio-demographic diversity in participants at baseline in the NutriNet-Santé study, which showed resemblance in terms of age and income distribution with the French general population [62]. However, caution is therefore needed when interpreting and generalizing results. Differences in nutrient intake between SEP categories are probably larger in the general population. Web-based design may affect internal validity by inducing misreporting. Regarding estimations of dietary intakes, studies investigating the validity of our web-based, self-reported dietary record tool against biomarkers showed the tool used in the NutriNet-Santé study performs well in estimating protein and potassium intakes and fairly well in estimating sodium intake but also provides reasonable estimates of true intake of fruits, vegetables, fish, beta carotene, vitamin C, and *n*-3 fatty acids [20,21]. A strength of our study was its reliance on at least three non-consecutive-day dietary records, which are recommended methods in large epidemiological studies [66], enabling a reliable estimation of usual diet [67]. The issue of accuracy of web-based self-reported data also arises for repeated 24-h dietary records compared to interviews by trained dietitians but previous work showed high agreement between the two methods in the case of our study [22]. Moreover, some inherent biases to studies based on interviews may be less pronounced in a web-based environment. In particular, bias associated with social desirability is probably lower in web-based studies due to higher perceived anonymity [22]. Regarding socioeconomic data, a previous study showed that the quality of information provided by the web-based socio-demographic questionnaires used in the study was equal to, or better than, that of the paper [27]. The large size of our sample may also have been a constraint, since significant results were found even when the difference in intake according to socioeconomic categories was small. We therefore interpreted as significant only those results for which the difference in nutrient intake between individuals with high and low SEP was >5%. In addition, the large size and the diversity of socio-demographic profiles through web-based questionnaires provided high statistical power to investigate stratified associations between income and nutrient intake by education and occupation categories. However, despite of this statistical power, some interactions remain unexplained. A limitation in our analysis is that the estimation of nutrient intakes did not include nutrients from supplements although supplement use is influenced by SEP [68]. This might have led to an underestimation of the observed associations between SEP and nutrient intake. Another limitation was that relative differences in intake between managerial and some categories of occupation were not interpreted since these groups are very heterogeneous in terms of social status and networks. These categories were those composed of individuals outside the paid workforce and also self-employed occupation class. Personal income is also a sensitive question and participants may overstate it or be reluctant to provide such information. Therefore, socioeconomic differentials may be misestimated [17]. In addition, as information regarding ethnicity or household relationships between participants was not available in our study, our models were not adjusted on these individual characteristics, which may be potential confounding factors.

## 5. Conclusions

Low socioeconomic groups had lower intakes than high socioeconomic groups for several nutrients, such as vitamins, minerals or fibre while no difference was found regarding nutrients that have recommended maximum cut offs, except from cholesterol. The use of education, income and occupation simultaneously and the study of interactions between these indicators allowed specific relationships to be highlighted according to the different socioeconomic indicators and consequently provide useful information to a better understanding of the mechanisms leading to social inequalities in health. Our study emphasizes that differences in nutrient intakes are likely to be the combined result of complex effects of the three different socioeconomic indicators in relation to diet. Among these effects, we discussed the affordability of nutrient-dense foods, the ability to understand health messages and to perform healthy dietary habits or the membership of a social network. Among them, education level appears to be an important driver of nutrient intake in low SEP groups. However, deeper investigations of the relationships between general level of education, nutrition knowledge, health literacy and dietary choices are needed. As nutritional interventions in at risk populations is a key element of public health policies, their implementation could be improved by a better knowledge of the socioeconomic indicators at stake for differences in nutrient intakes between individuals. Further studies assessing the dynamic nature of socioeconomic indicators, using repeated measures during the life course would be useful to provide a better understanding of their cumulative effects on nutrient intake and consequently, of the long-term health impact.

## Figures and Tables

**Table 1:** Socioeconomic and socioeconomic characteristics of the sample (*N* = 91,900).

Variables	Women	Men	*p* *
	*N* = 72,154	*N* = 19,746	
**Age class**			<0.0001
18–30	25.6	14.8	
30–50	42.0	36.4	
50–65	27.7	35.0	
>65	4.7	13.8	
**Educational level**			<0.0001
Primary	2.6	3.5	
Secondary	33.6	33.9	
Undergraduate	32.5	24.4	
Postgraduate	31.3	38.2	
**Occupational category**			<0.0001
Never employed	6.1	3.2	
Manual worker	2.0	5.2	
Employee	34.0	13.8	
Intermediate profession	27.0	23.4	
Self-employed	2.8	4.8	
Managerial staff	28.1	49.6	
**Household income per consumption unit**			<0.0001
<1200 euros	18.4	12.5	
1200–1800 euros	25.1	23.0	
1800–2700 euros	22.6	25.7	
>2700 euros	21.0	31.9	
Not answered	12.9	6.9	

* *p*-value for chi square analysis.

**Table 2:** Differences in nutrient intake between the highest and the lowest socioeconomic position (SEP) categories of occupation, household income and education in women (*N* = 72,154) ^1^.

Nutrients	Mean (SD) in Total Sample	Occupational Category			Household Income per Unit and per Month			Education Level		
		Difference between Managerial Staff and Manual Workers ^2^	Relative Difference ^3^	*p*-Value	Difference between >2700 € and <1200 € ^2^	Relative Difference ^3^	*p*-Value	Difference between Postgraduate and Primary Level ^2^	Relative Difference ^3^	*p*-Value
Total energy (kcal/day)	1800.0 (435)	−40.0	−2.3%	0.0005	19.1	1.1%	0.0004	105.2	5.8%	<0.0001
Proteins (g/day)	75.9 (20)	0.5	0.7%	<0.0001	1.7	2.2%	0.003	−4.4	−5.9%	<0.0001
Total carbohydrates (g/day)	187.8 (54.1)	−3.5	−1.9%	<0.0001	−3.8	−2.1%	0.001	6.0	3.1%	<0.0001
Complex carbohydrates (g/day)	96.8 (33.9)	−3.5	−3.8%	<0.0001	−5.2	−5.6%	<0.0001	2.4	2.5%	<0.0001
Sugars (g/day)	90.4 (32.8)	−0.07	−0.1%	<0.0001	1.4	1.5%	0.003	3.6	4.0%	<0.0001
Fibre (g/day)	18.8 (6.9)	0.8	4.2%	<0.0001	0.5	2.8%	0.0007	1.6	7.7%	<0.0001
Lipids (g/day)	77.6 (25.4)	0.03	0.0%	<0.0001	−0.5	−0.6%	0.3	−0.8	−1.1%	0.005
Polyunsaturated fatty acids (g/day)	11.0 (5.1)	−0.07	–0.6%	0.4	0.0	−0.3%	0.4	−0.3	−2.6%	0.0003
Monounsaturated fatty acids (g/day)	29.0 (10.5)	0.6	2.0%	<0.0001	0.1	0.2%	0.6	−0.3	−0.9%	0.5
Saturated fatty acids (g/day)	31.8 (12.2)	−0.5	−1.7%	<0.0001	−0.6	−2.0%	0.05	−0.2	−0.7%	0.3
Cholesterol (mg/day)	306.1 (136.1)	−0.9	−0.3%	<0.0001	2.5	0.8%	0.4	−22.4	−7.6%	<0.0001
Calcium (mg/day)	907.4 (301.4)	29.5	3.2%	<0.0001	18.1	2.0%	0.0004	4.7	0.5%	<0.0001
Iron (mg/day)	12.9 (4.6)	0.4	3.0%	<0.0001	0.2	1.4%	0.3	0.4	3.2%	<0.0001
Magnesium (mg/day)	321.7 (105.3)	21.5	6.5%	<0.0001	20.2	6.0%	<0.0001	9.0	2.7%	<0.0001
Potassium (mg/day)	2882.6 (785.4)	72.4	2.4%	<0.0001	122.5	4.1%	<0.0001	20.0	0.7%	<0.0001
Sodium (g/day)	2484.8 (807.5)	48.8	2.0%	0.0009	30.2	1.2%	0.6	−70.3	−2.9%	0.002
Vitamin A (µg/day)	1040.5 (824.3)	24.4	2.2%	<0.0001	44.5	4.0%	0.2	18.8	1.7%	0.2
Beta carotene (µg/day)	3409.2 (2653)	253.9	7.1%	<0.001	256.4	7.1%	0.02	340.3	9.2%	<0.0001
Folate (µg/day)	321.7 (115.7)	13.5	4.1%	<0.0001	17.9	5.3%	<0.0001	12.8	3.8%	<0.0001
Vitamin C (mg/day)	117.2 (84.9)	4.0	3.4%	<0.0001	14.8	11.8%	<0.0001	11.2	9.0%	<0.0001
Vitamin D (µg/day)	2.6 (2.2)	0.3	11.5%	<0.0001	0.3	9.9%	0.004	−0.1	−3.4%	0.03
Vitamin E (mg/day)	11.3 (4.8)	0.2	2.1%	0.6	0.1	0.5%	0.3	−0.2	1.8%	0.3
Alcohol (g/day) ^4^	10.5 (10.9)	1.8	15.6%	<0.0001	0.4	3.3%	0.01	−0.4	−3.6%	0.0004

^1^ All models for food nutrients intake were adjusted for age, total energy intake and the three SEP indicators; ^2^ Subtraction of the mean intake between individuals belonging to the highest SEP category and those of the lowest category; ^3^ Relative difference in mean intake between individuals belonging to the highest SEP category and those of the lowest category was computed as ((mean intake of the highest SEP category − mean intake of the lowest SEP category)/(mean intake of the highest SEP category)) × 100). A positive relative difference indicates a higher intake in high SEP participants. A negative relative difference indicates a lower intake in high SEP participants; ^4^ Mean in consumers only.

**Table 3:** Differences in nutrient intake between the highest and the lowest SEP categories of occupation, household income and education in men (*N* = 19,746) ^1^.

Nutrients	Mean (SD) in Total Sample	Occupational Category			Household Income per Unit and per Month			Education Level		
		Difference between Managerial Staff and Manual Worker ^2^	Relative Difference ^3^	*p*-Value	Difference between >2700 € and <1200 € ^2^	Relative Difference ^3^	*p*-Value	Difference between Postgraduate and Primary Level ^2^	Relative Difference ^3^	*p*-Value
Total energy (kcal/day)	2274.8 (542)	−79.7	−3.6%	0.0007	24.4	1.1%	0.08	19.7	0.9%	0.4
Proteins (g/day)	94.6 (25.3)	−1.9	−2.0%	0.3	2.6	2.7%	0.10	−4.8	−5.1%	<0.0001
Total carbohydrates (g/day)	234.3 (68.1)	−7.5	−3.2%	<0.0001	−4.3	−1.8%	0.1	9.2	3.8%	<0.0001
Complex carbohydrates (g/day)	129.2 (44.7)	−4.3	−3.4%	0.002	−7.9	−6.3%	0.0003	2.1	1.6%	0.4
Sugars (g/day)	104.4 (40.2)	−3.2	−3.1%	<0.0001	3.5	3.3%	0.05	7.0	6.6%	<0.0001
Fibre (g/day)	22.2 (8.5)	−0.4	−1.9%	0.09	0.2	0.7%	0.1	1.6	6.9%	<0.0001
Lipids (g/day)	94.2 (30.2)	1.8	1.9%	0.07	−0.9	−0.9%	0.9	−0.9	−0.9%	0.5
Polyunsaturated fatty acids (g/day)	13.6 (6.5)	0.0	−0.2%	0.5	−0.6	−4.2%	0.2	0.0	−0.1%	0.2
Monounsaturated fatty acids (g/day)	35.2 (12.4)	0.6	1.6%	0.02	−0.1	−0.3%	0.9	−0.4	−1.1%	0.2
Saturated fatty acids (g/day)	38.4 (14.5)	1.1	2.8%	0.1	−0.2	−0.5%	0.7	−0.2	−0.6%	0.6
Cholesterol (mg/day)	375.4 (167.3)	7.3	1.9%	0.01	0.6	0.2%	0.8	−33.5	−9.2%	<0.0001
Calcium (mg/day)	1036.4 (360)	3.6	0.4%	0.002	28.4	2.7%	0.1	10.4	1.0%	0.002
Iron (mg/day)	15.9 (5.9)	−0.4	−2.4%	0.0003	0.3	1.7%	0.7	0.3	2.0%	<0.0001
Magnesium (mg/day)	388.0 (128.4)	1.3	0.3%	0.03	26.0	6.6%	<0.0001	8.5	2.2%	<0.0001
Potassium (mg/day)	3406.4 (914)	−39.7	−1.2%	0.8	8.6	5.3%	<0.0001	21.9	0.6%	<0.0001
Sodium (g/day)	3191.7 (1050)	17.3	0.5%	0.3	−47.3	−1.5%	0.7	−130.1	−4.2%	0.0004
Vitamin A (µg/day)	1198.8 (989)	32.2	2.6%	0.2	8.6	0.7%	0.8	−23.7	−1.9%	0.3
Beta carotene (µg/day)	3600.2 (2932)	90.7	2.6%	0.4	217.0	5.9%	0.1	228.6	6.0%	<0.0001
Folate (µg/day)	365.2 (132.6)	−7.2	−2.0%	0.4	19.4	5.2%	0.002	11.4	3.0%	<0.0001
Vitamin C (mg/day)	126.6 (83.7)	3.3	2.6%	0.07	17.9	13.6%	0.0003	8.9	6.8%	0.0007
Vitamin D (µg/day)	3.2 (2.7)	0.1	6.7%	0.03	0.3	9.0%	0.1	0.1	3.1%	0.2
Vitamin E (mg/day)	13.0 (5.7)	−0.2	−1.9%	0.2	0.0	−0.3%	0.09	0.3	2.0%	0.07
Alcohol (g/day) ^4^	20.2 (18.7)	1.8	8.6%	<0.0001	2.0	9.4%	0.002	−1.5	−8.2%	0.04

^1^ All models for nutrients intake were adjusted for age, total energy intake and the three SEP indicators; ^2^ Subtraction of the mean intake between individuals belonging to the highest SEP category and those of the lowest category; ^3^ Relative difference in mean intake between individuals belonging to the highest SEP category and those of the lowest category was computed as ((mean intake of the highest SEP category − mean intake of the lowest SEP category)/(mean intake of the highest SEP category)) × 100). A positive relative difference indicates a higher intake in high SEP participants. A negative relative difference indicates a lower intake in high SEP participants; ^4^ Mean in consumers only.

**Table 4:** Associations between dietary intake of magnesium, folate and beta-carotene and income stratified by educational level in women (*N* = 72,154) ^1,4^.

Nutrients	Incomes (€) per Person per Month	Primary					Secondary					Undergraduate					Postgraduate				
		Mean	SE ^5^	Difference with >2.700 € ^2^	Relative Difference (%) ^3^	*p*	Mean	SE ^5^	Difference with >2.700 € ^2^	Relative Difference (%) ^3^	*p*	Mean	SE ^5^	Difference with >2.700 € ^2^	Relative Difference (%) ^3^	*p*	Mean	SE ^5^	Difference with >2.700 € ^2^	Relative Difference (%) ^3^	*p*
Magnesium	<1200	292.2	4.9	32.1	9.9%	0.001	303.8	1.4	19.9	6.2%	<0.0001	321.3	2.0	13.9	4.1%	<0.0001	336.4	2.7	9.4	2.7%	<0.0001
	1200–1800	308.9	5.0	15.4	4.7%		310.3	1.4	13.4	4.1%		323.0	1.9	12.1	3.6%		334.1	2.6	11.7	3.4%	
	1800–2700	303.4	6.3	20.8	6.4%		321.0	1.6	2.7	0.8%		329.5	1.9	5.6	1.7%		340.0	2.5	5.8	1.7%	
	>2700	324.3	8.4	0.0	0.0%		323.7	1.9	0.0	0.0%		335.1	2.0	0.0	0.0%		345.8	2.4	0.0	0.0%	
Folate	<1200	292.9	5.7	13.9	4.5%	0.09	307.6	1.6	21.5	6.5%	<0.0001	321.9	2.3	15.0	4.5%	<0.0001	336.6	3.1	11.2	3.2%	<0.0001
	1200–1800	309.4	5.9	−2.6	-0.8%		318.7	1.6	10.3	3.1%		325.1	2.1	11.9	3.5%		333.5	3.0	14.3	4.1%	
	1800–2700	302.9	7.4	3.9	1.3%		324.8	1.8	4.3	1.3%		332.5	2.2	4.4	1.3%		339.3	2.9	8.5	2.5%	
	>2700	306.8	9.9	0.0	0.0%		329.1	2.2	0.0	0.0%		336.9	2.3	0.0	0.0%		347.8	2.8	0.0	0.0%	
Beta-carotene	<1200	3041.1	135.5	105.5	3.4%	0.06	3209.3	39.3	219.4	6.4%	<0.0001	3419.4	56.9	164.8	4.6%	0.08	3725.2	81.2	−24.0	−0.7%	0.03
	1200–1800	3372.4	139.0	−225.7	−7.2%		3289.1	39.0	139.7	4.1%		3391.0	53.5	193.2	5.4%		3560.2	76.1	141.1	3.8%	
	1800–2700	3097.9	175.6	48.7	1.6%		3446.0	44.4	−17.3	−0.5%		3432.4	54.9	151.9	4.2%		3722.3	75.1	−21.1	−0.6%	
	>2700	3146.7	233.8	0.0	0.0%		3428.8	54.1	0.0	0.0%		3584.2	58.6	0.0	0.0%		3701.2	72.7	0.0	0.0%	

^1^ All models for nutrients intake were adjusted for age, total energy intake and occupation; ^2^ Subtraction of the mean intake between individuals belonging to the highest income category and those of the lowest income category; ^3^ Relative difference in mean intake between individuals belonging to the highest SEP category and those of the lowest category was computed as ((mean intake of the highest SEP category − mean intake of the lowest SEP category)/(mean intake of the highest SEP category)) × 100). A positive relative difference indicates a higher intake in high SEP participants. A negative relative difference indicates a lower intake in high SEP participants; ^4^ The category of income corresponding to individuals who refused to answer was not presented in table as it was not interpreted, although it was included in the models; ^5^ Standard error.

**Table 5:** Associations between dietary intake of magnesium, potassium, folate, beta-carotene, proteins, fibre and income stratified by educational level in men (*N* = 19,746) ^1,4^.

Nutrients	Incomes (€) per Person per Month	Primary					Secondary					Undergraduate					Postgraduate				
		Mean	SE ^5^	Difference with >2700 € ^2^	Relative Difference (%) ^3^	*p*	Mean	SE ^5^	Difference with >2700 € ^2^	Relative Difference (%) ^3^	*p*	Mean	SE ^5^	Difference with >2700 € ^2^	Relative Difference (%) ^3^	*p*	Mean	SE ^5^	Difference with >2700 € ^2^	Relative Difference (%) ^3^	*p*
Magnesium	<1200	350.7	13.1	51.6	12.8%	0.0003	357.7	3.3	29.8	7.7%	<0.0001	382.6	4.6	10.8	2.8%	0.0038	388.0	5.8	16.7	4.1%	<0.0001
	1200–1800	366.7	11.5	35.6	8.9%		368.1	2.8	19.4	5.0%		379.5	3.9	14.0	3.6%		391.1	5.1	13.6	3.4%	
	1800–2700	363.3	12.7	38.9	9.7%		383.8	3.0	3.7	0.9%		389.4	4.1	4.1	1.0%		390.7	4.8	14.0	3.5%	
	>2700	402.3	14.4	0.0	0.0%		387.5	3.6	0.0	0.0%		393.5	4.3	0.0	0.0%		404.7	4.6	0.0	0.0%	
Potassium	<1200	3147.4	87.3	217.2	6.5%	0.009	3239.8	22.0	175.5	5.1%	<0.0001	3370.9	31.1	61.9	1.8%	0.001	3358.2	19.7	113.0	3.3%	0.0005
	1200–1800	3173.9	76.4	190.7	5.7%		3330.5	18.3	84.9	2.5%		3346.4	26.4	86.4	2.5%		3396.0	18.4	75.2	2.2%	
	1800–2700	3198.6	84.6	166.0	4.9%		3381.8	20.1	33.5	1.0%		3442.0	27.5	−9.2	−0.3%		3421.9	18.2	49.2	1.4%	
	>2700	3364.6	96.1	0.0	0.0%		3415.3	24.1	0.0	0.0%		3432.8	28.9	0.0	0.0%		3471.2	17.6	0.0	0.0%	
Folate	<1200	339.6	14.3	11.9	3.4%	0.04	341.1	3.2	29.4	7.9%	<0.0001	362.7	4.9	12.7	3.4%	0.009	369.0	6.4	9.0	2.4%	0.006
	1200–1800	333.5	12.5	18.0	5.1%		355.3	2.9	15.3	4.1%		363.5	4.2	12.0	3.2%		365.4	5.6	12.6	3.3%	
	1800–2700 €	352.9	13.8	−1.3	−0.4%		358.7	3.2	11.8	3.2%		372.7	4.3	2.7	0.7%		366.8	5.3	11.2	3.0%	
	>2700	351.5	15.7	0.0	0.0%		370.5	3.9	0.0	0.0%		375.4	4.6	0.0	0.0%		378.1	5.1	0.0	0.0%	
Beta-carotene	<1200	3207.5	335.1	261.5	7.5%	0.2	3176.0	84.9	462.4	12.7%	0.002	3676.4	128.9	−2.9	−0.1%	0.04	3621.7	156.1	128.7	3.4%	0.07
	1200–1800	3059.7	293.2	409.3	11.8%		3407.9	70.7	230.5	6.3%		3495.7	109.6	177.9	4.8%		3584.6	136.6	165.8	4.4%	
	1800–2700	3371.1	324.4	97.9	2.8%		3563.3	77.6	75.1	2.1%		3705.5	114.0	−32.0	−0.9%		3648.8	128.9	101.6	2.7%	
	>2700	3469.0	368.6	0.0	0.0%		3638.4	99.8	0.0	0.0%		3673.6	119.9	0.0	0.0%		3750.4	123.1	0.0	0.0%	
Proteins	<1200	95.5	2.3	2.1	2.1%	0.008	94.3	0.6	0.2	0.3%	0.4	94.5	0.8	−0.1	−0.1%	0.9	91.8	1.0	1.5	1.6%	0.05
	1200–1800	90.0	2.0	7.6	7.8%		95.5	0.5	−1.0	−1.0%		94.4	0.7	0.0	0.0%		92.2	0.8	1.1	1.2%	
	1800–2700	92.3	2.2	5.3	5.4%		94.9	0.5	−0.4	−0.4%		95.1	0.7	−0.6	−0.7%		92.3	0.8	0.9	1.0%	
	>2700	97.6	2.5	0.0	0.0%		94.6	0.6	0.0	0.0%		94.4	0.7	0.0	0.0%		93.3	0.8	0.0	0.0%	
Fibre	<1200	21.3	0.8	−0.6	−2.7%	0.03	20.9	0.2	1.4	6.3%	0.0001	22.8	0.3	−0.3	−1.5%	0.2	23.8	0.4	−0.6	−2.5%	0.5
	1200–1800	20.7	0.7	0.1	0.3%		21.5	0.2	0.8	3.7%		22.1	0.3	0.4	1.6%		23.2	0.4	0.1	0.3%	
	1800–2700	21.0	0.8	−0.3	−1.2%		22.1	0.2	0.2	0.8%		22.6	0.3	−0.1	−0.4%		23.2	0.3	0.0	0.0%	
	>2700	20.7	0.9	0.0	0.0%		22.3	0.3	0.0	0.0%		22.5	0.3	0.0	0.0%		23.2	0.3	0.0	0.0%	

^1^ All models for nutrients intake were adjusted for age, total energy intake and occupation; ^2^ Subtraction of the mean intake between individuals belonging to the highest income category and those of the lowest income category;^3^ Relative difference in mean intake between individuals belonging to the highest SEP category and those of the lowest category was computed as ((mean intake of the highest SEP category − mean intake of the lowest SEP category)/(mean intake of the highest SEP category)) × 100). A positive relative difference indicates a higher intake in high SEP participants. A negative relative difference indicates a lower intake in high SEP participants; ^4^ The category of income corresponding to individuals who refused to answer was not presented in table as it was not interpreted, although it was included in the models; ^5^ Standard error.

**Table 6:** Associations between dietary intake of complex carbohydrates, magnesium, potassium and alcohol and income stratified by occupational level in women (*N* = 72,154) or men (*N* = 19,746) ^1,4^.

Nutrients	Incomes per Person per Month (€)	Manual Workers					Employees					Intermediate Profession					Managerial Staff				
		Mean	SE ^5^	Difference with >2700 € ^2^	Relative Difference (%) ^3^	*p*	Mean	SE ^5^	Difference with >2700 € ^2^	Relative Difference (%) ^3^	*p*	Mean	SE ^5^	Difference with >2700 € ^2^	Relative Difference (%) ^3^	*p*	Mean	SE ^5^	Difference with >2700 € ^2^	Relative Difference (%) ^3^	*p*
**Women**																					
Complex carbohydrates	<1200	99.1	1.7	−8.0	−8.79%	0.5	97.4	0.4	−4.0	−4.2%	<0.0001	99.0	0.7	−5.3	−5.6%	<0.0001	95.5	1.0	−3.0	−3.2%	<0.0001
	1200–1800	99.1	1.7	−8.0	−8.8%		96.1	0.4	−2.6	−2.8%		97.1	0.6	−3.4	−3.6%		95.9	0.8	−3.4	−3.7%	
	1800–2700	97.9	2.6	−6.8	−7.5%		94.4	0.5	−1.0	−1.0%		94.4	0.5	−0.8	−0.8%		93.2	0.7	−0.7	−0.8%	
	>2700	91.1	4.8	0.0	0.0%		93.5	0.6	0.0	0.0%		93.7	0.6	0.0	0.0%		92.5	0.7	0.0	0.0%	
Magnesium	<1200	304.5	5.7	2.0	0.7%	0.2	310.2	1.5	21.5	6.5%	<0.0001	327.2	2.5	11.1	3.3%	<0.0001	326.5	3.6	12.8	3.8%	<0.0001
	1200–1800	316.5	5.9	−10.0	−3.3%		314.5	1.3	17.2	5.2%		327.8	2.0	10.5	3.1%		327.2	2.8	12.1	3.6%	
	1800–2700	318.5	9.0	−11.9	−3.9%		322.1	1.6	9.6	2.9%		333.1	2.0	5.2	1.5%		335.8	2.6	3.6	1.1%	
	>2700	306.5	16.4	0.0	0.0%		331.7	2.2	0.0	0.0%		338.3	2.2	0.0	0.0%		339.3	2.5	0.0	0.0%	
**Men**																					
Magnesium	<1200	372.7	8.4	37.8	9.2%	0.02	370.0	4.9	33.7	8.4%	0.003	369.7	5.1	15.3	4.0%	0.004	367.5	3.6	28.4	7.2%	<0.0001
	1200–1800	384.0	8.1	26.5	6.5%		373.7	4.2	30.0	7.4%		370.7	3.6	14.4	3.7%		381.0	2.8	14.9	3.8%	
	1800–2700	391.0	11.0	19.5	4.8%		379.2	4.8	24.5	6.1%		381.0	3.5	4.0	1.0%		387.6	2.6	8.4	2.1%	
	>2700	410.5	19.2	0.0	0.0%		403.7	7.9	0.0	0.0%		385.0	4.6	0.0	0.0%		395.9	2.5	0.0	0.0%	
Potassium	<1200	3284.4	57.8	423.0	11.4%	0.0002	3311.5	34.4	217.8	6.2%	0.003	3344.0	34.3	63.2	1.9%	0.02	3286.7	38.9	166.7	4.8%	<0.0001
	1200–1800	3379.1	55.8	328.3	8.9%		3341.1	29.2	188.2	5.3%		3333.1	24.1	74.1	2.2%		3387.8	23.2	65.6	1.9%	
	1800-2700	3331.4	75.8	376.0	10.1%		3407.6	33.8	121.7	3.5%		3410.3	23.6	−3.2	-0.1%		3427.0	19.7	26.4	0.8%	
	>2700	3707.4	131.7	0.0	0.0%		3529.3	55.0	0.0	0.0%		3407.2	30.4	0.0	0.0%		3453.4	18.6	0.0	0.0%	
Alcohol	<1200	20.0	1.8	2.6	11.5%	0.4	17.6	1.1	0.8	4.1%	0.7	20.6	1.0	−1.3	−6.7%	0.5	21.1	1.2	1.4	6.0%	0.008
	1200–1800	20.7	1.7	2.0	8.7%		19.2	0.9	−0.8	−4.3%		19.8	0.7	−0.5	−2.6%		20.2	0.7	2.3	10.1%	
	1800–2700	19.8	2.3	2.8	12.4%		17.9	1.0	0.4	2.3%		19.6	0.6	−0.3	−1.4%		21.6	0.6	0.8	3.7%	
	>2700	22.6	4.2	0.0	0.0%		18.4	1.5	0.0	0.0%		19.3	0.8	0.0	0.0%		22.5	0.5	0.0	0.0%	

^1^ All models for nutrients intake were adjusted for age, total energy intake and education; ^2^ Subtraction of the mean intake (g/day) between individuals belonging to the highest income category and those of the lowest income category; ^3^ Relative difference in mean intake between individuals belonging to the highest income category and those of the lowest income category was computed as ((mean intake of the highest income category − mean intake of the lowest income category)/(mean intake of the highest income category)) × 100). A positive relative difference indicates a higher intake in high SEP participants. A negative relative difference indicates a lower intake in high SEP participants; ^4^ The category of income corresponding to individuals who refused to answer was not presented in table as it was not interpreted, although it was included in the models. ^5^ Never employed and self-employed categories were not presented in the table as they were not interpreted, although they were included in the models; **^5^** Standard error.

## Abbreviations

The following abbreviations are used in this manuscript:

SEP: SocioEconomic Position
DEDIPAC: Determinants of Diet and Physical Activity
BMR: Basal Metabolic Rate
